# Supplementary material for: Prophage exotoxins enhance colonization fitness in epidemic scarlet fever-causing Streptococcus pyogenes
Source: Nat Commun. 2020 Oct 6;11:5018. doi: 10.1038/s41467-020-18700-5 (PMC7538557; doi:10.1038/s41467-020-18700-5)
Supplement: Supplementary file 1 — Supplementary Information [file 41467_2020_18700_MOESM1_ESM.pdf]

Supplementary Information

Prophage exotoxins enhance colonization fitness in epidemic scarlet fever-causing *Streptococcus pyogenes*

Brouwer et al.

Methods

Construction of reporter strains

The plasmid-based reporter system (pLZ12Km2-P23R:TA, Addgene plasmid gift from Associate Professor Thomas Proft, University of Auckland, New Zealand) described in[1] was used to construct plasmid pLZ12Km2-P23R:TA:GFP. Maintenance plasmid pUC57-RBSGFP containing the ribosomal binding site (RBS) and *gfp* gene from pDCerm-GFP[2] was synthesized commercially by Genscript. pLZ12Km2-P23R:TA was digested with *Not*I, and pUC57-RBSGFP was incubated with *Not*I to excise the RBS and *gfp* gene from pUC57-RBSGFP. The excised RBS and *gf*p were then ligated into digested pLZ12km2-P23R:TA to generate pLZ12Km2-P23R:TA:GFP, which was used for transformation of electrocompetent HKU16 cells.

Supplementary Figures

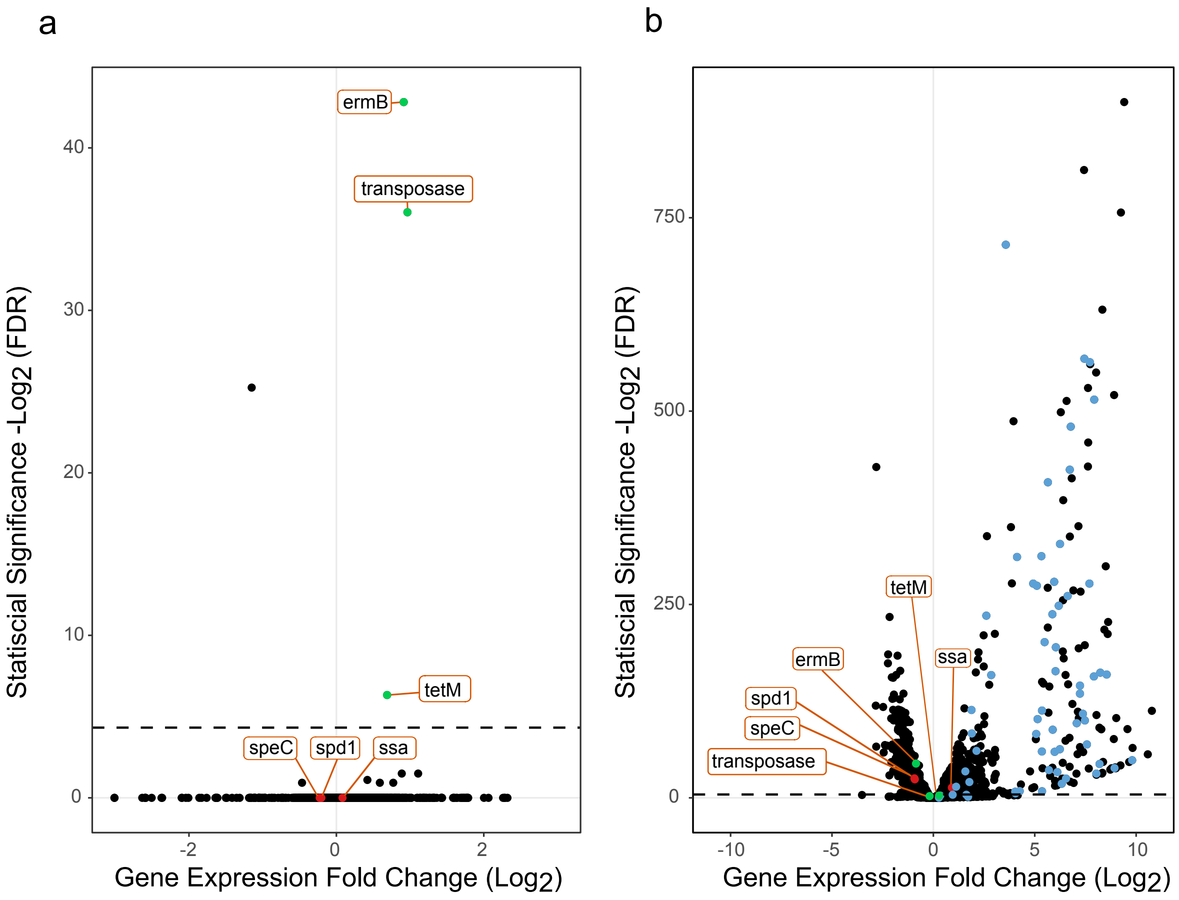

Supplementary Figure 1: Global transcriptional changes in HKU16 in response to antibiotic treatment stress. RNA-seq transcriptomes of *S. pyogenes emm*12 strain HKU16 grown in THY medium compared with (**a**) THY supplemented with 2 µg/ml erythromycin and (**b**) THY

supplemented with 0.2 µg/ml mitomycin C. Volcano plots represent differentially expressed genes of erythromycin or mitomycin C supplemented cultures relative to cultures grown in THY medium alone. Each dot represents a gene expression fold change (horizontal axis) with respect to statistical significance (vertical axis). Genes relating to prophage ϕHKU.vir virulence factors (red dots) and ICE-HKUe*mm*12 genes (green dots) are annotated as indicated using orange boxes. Genes corresponding to prophage ΦHKU.vir genes are colored blue in (**b**). Dashed line indicates a false discovery rate (FDR) of $-Log_2$ 0.05.

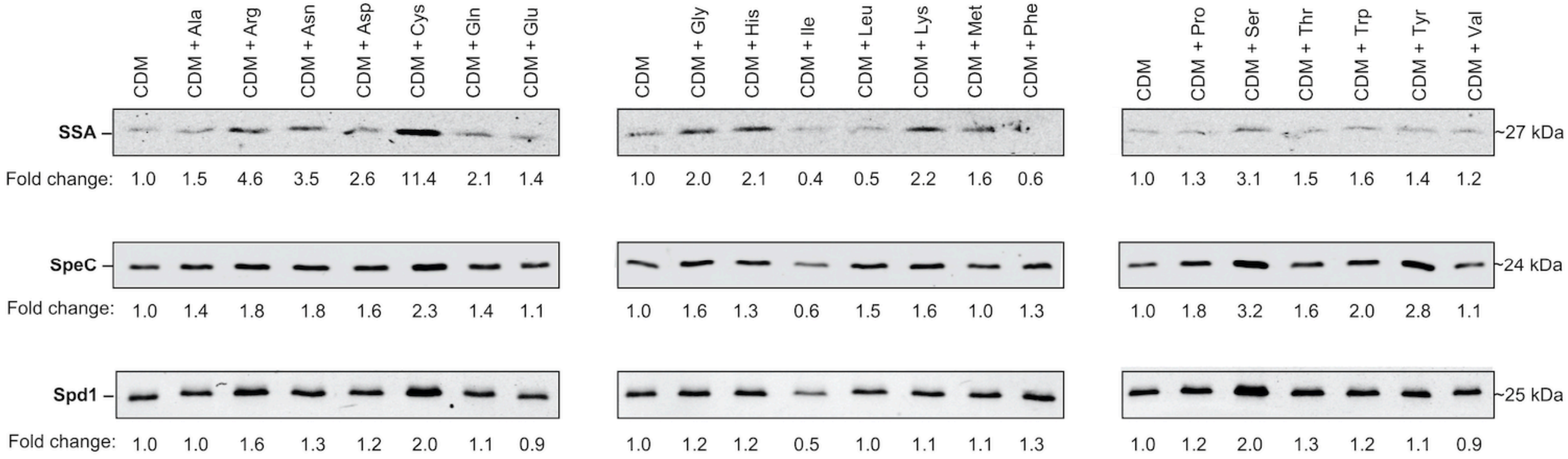

Supplementary Figure 2: Small molecule screen of all 20 amino acids used to identify cysteine as a factor specifically enhancing release of the exotoxin SSA by HKU16 grown in chemically defined medium (CDM) (n = 1). Conditions with a value greater than or equal to a cut-off value of 5-fold were selected for further analyses. Western blot signal intensities were quantified with ImageJ. Source data are provided as a Source Data file.

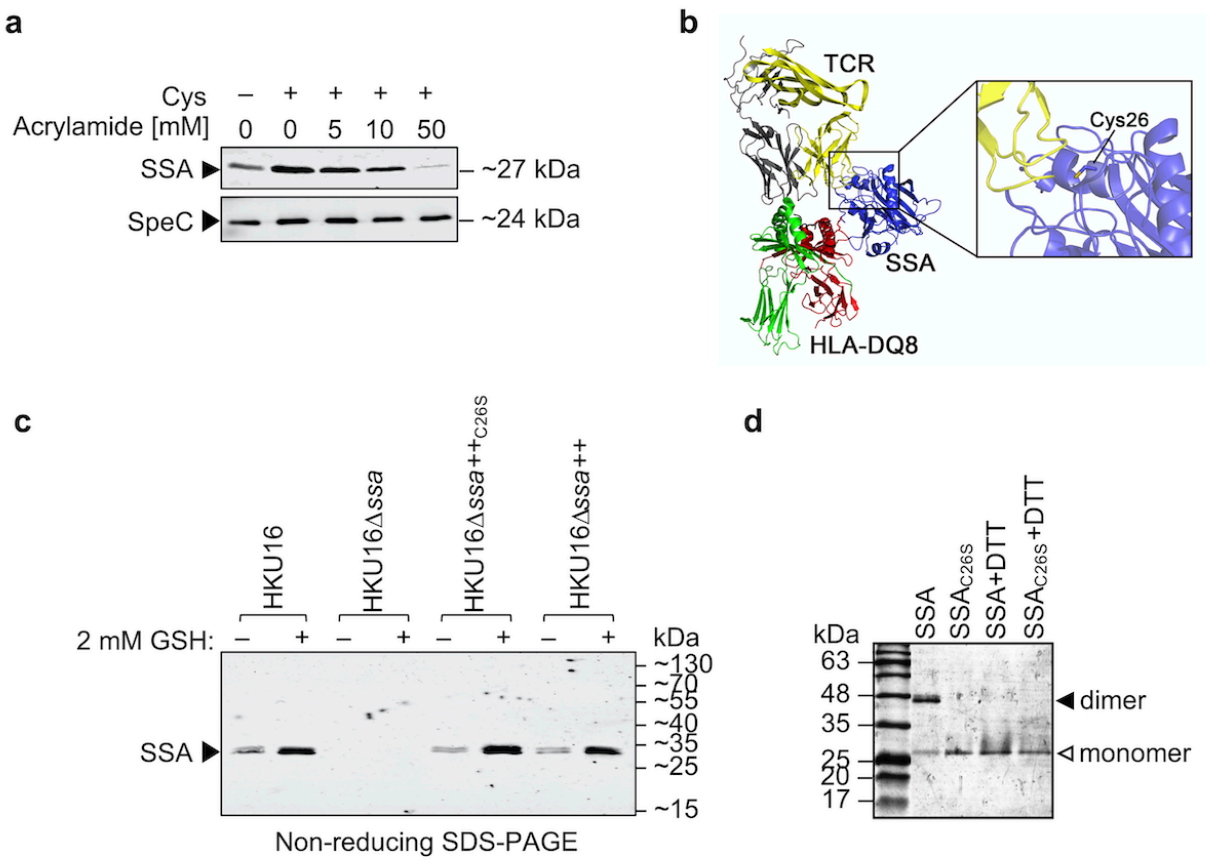

Supplementary Figure 3: Thiol-mediated regulation of the superantigen SSA. (**a**) Western immunoblot detection of secreted SSA and SpeC after supplementation of CDM with 2 mM Cys pre-treated with increasing concentrations of acrylamide (n = 1). (**b**) Ribbon diagram representation of the modelled SSA-mediated T cell activation complex. The model was generated by superposition of the SSA (PDB 1BXT)[3] and HLA-DQ8 (PDB 1JK8)[4] crystal structures onto the co-crystal of SpeA in complex with TCRβ (PDB 1L0Y) and the co-crystal of SEB in complex with HLA-DR4

(PDB 1SEB)[5]. Colors are as follows: SSA, *blue*; TCR β-chain *yellow*; TCR α-chain *grey*; MHC α-chain, *red*; MHC β-chain, *green*. The inset highlights the location of the free Cys26 within the TCR-SSA interface. The image was generated using the PyMOL Molecular Graphics System, version 1.3 Schrödinger (www.pymol.org/). (**c**) Immunoblot detection of SSA secreted by indicated HKU16 strains grown in CDM supplemented with 2 mM of GSH following non-reducing SDS-PAGE (n = 1). (**d**) PAGE profile (lacking SDS and samples were not boiled) of purified WT SSA and mutant SSA_{C26S} under non-reducing and reducing conditions, respectively (n =1). Source data are provided as a Source Data file.

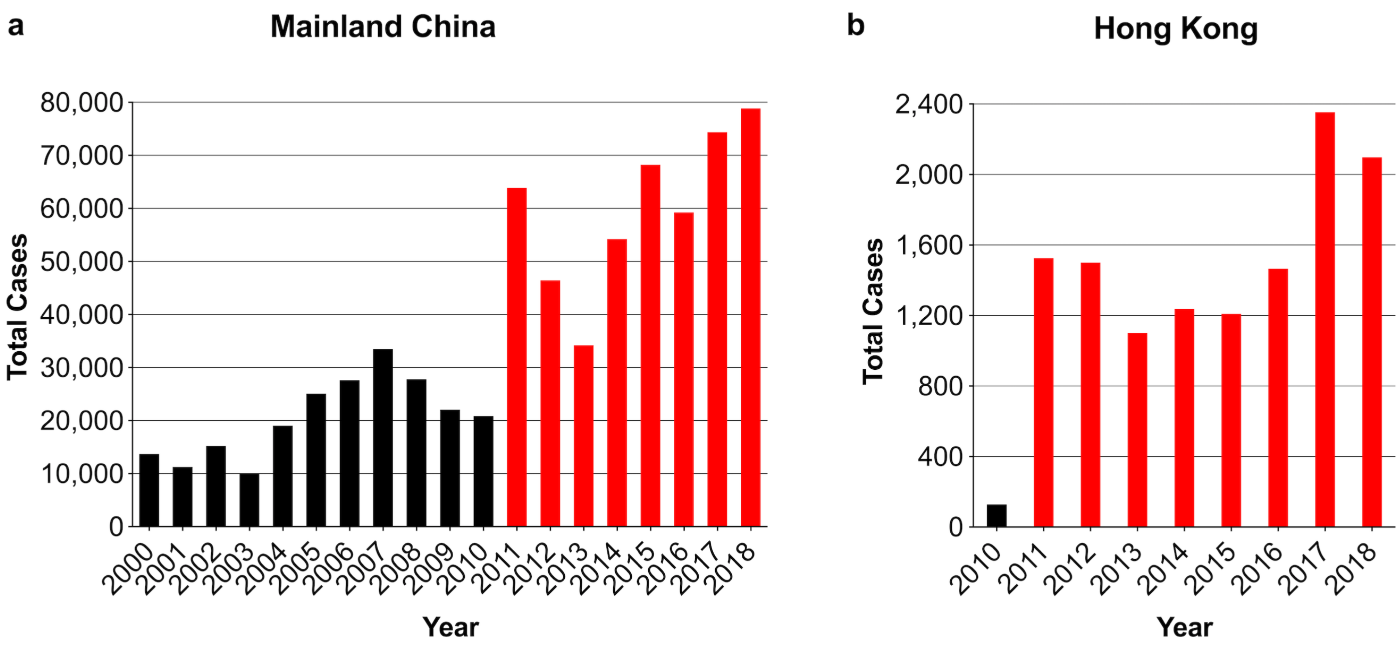

Supplementary Figure 4: Annual reported scarlet fever cases in (**a**) Mainland China and (**b**) Hong Kong. Data were obtained from the National Bureau of Statistics of China (http://data.stats.gov.cn; accessed August 20, 2019), and from the Hong Kong Centre for Health Protection website (http://www.chp.gov.hk/en/notifiable1/10/26/43.html; August 20, 2019), respectively. Ongoing resurgence of scarlet fever is highlighted in red.

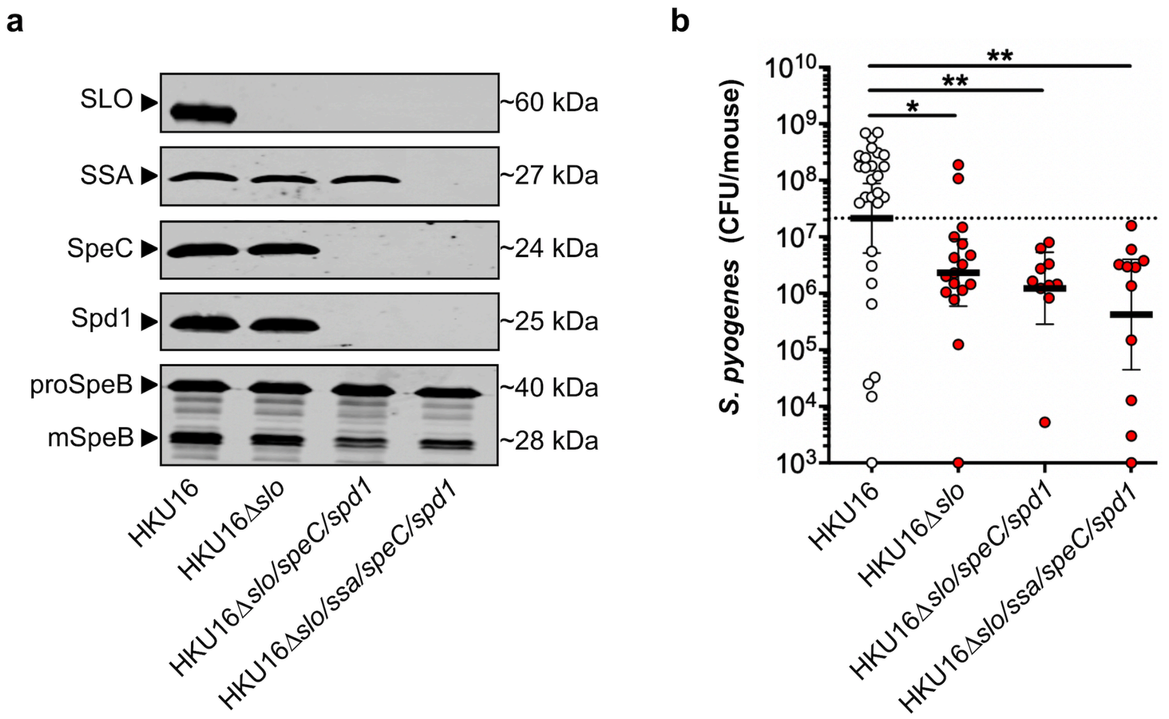

Supplementary Figure 5: Effect of combinations of SLO and ΦHKU.vir-encoded exotoxin mutations on the nasopharyngeal colonization fitness of HKU16. (**a**) Immunoblot detection of SLO, SSA, SpeC, Spd1 and SpeB expression from indicated HKU16 strains. The molecular mass of each protein (kDa) is indicated to the right. Protein levels of the 40-kDa zymogen form (proSpeB) and 28-kDa mature form of SpeB (mSpeB) are shown as loading control. (**b**) Individual 'humanized' B6 mice that express HLA-DR4, HLA-DQ8 and CD4 were nasally inoculated with $\sim 1 \times 10^8$ bacterial colony forming units (CFU) with indicated HKU16 strains and nasopharyngeal CFUs were assessed at 48 h post-infection. Data for wildtype HKU16 and HKU16Δ*slo* were taken from Figure 4d. Each symbol represents CFUs from an individual mouse ($n \geq 10$). Presented is the

geometric mean with 95% confidence interval. Significance was calculated using the Kruskal-Wallis test with the Dunn's multiple comparisons post-hoc test against the HKU16 control group (**p=0.0205 for HKU16Δ*slo*, **p=0.0097 for HKU16Δ*slo*/*speC*/*spd*1, and **p=0.003 for HKU16Δ*slo*/*ssa*/*speC*/*spd*1++). HKU16Δ*slo/speC/spd1* and HKU16Δ*slo/ssa/speC/spd1* showed no significant difference in colonization fitness compared to HKU16Δ*slo*. Source data are provided as a Source Data file.

Supplementary Table 1: List of bacterial strains, plasmids and primers used in this study.

Bacterial strains	Description	Reference/Source
E. coli		
MC1061	Laboratory cloning strain	[6]
XL1-blue	Laboratory cloning strain	Stratagene
BL21(DE3)	Laboratory expression strain	Stratagene
S. pyogenes		
HKU16	Hong Kong *S. pyogenes emm12* scarlet fever isolate	[7]
HKU16Δ*ssa*	HKU16Δ*ssa* isogenic mutant strain	This study
HKU16Δ*ssa*++	HKU16Δ*ssa*::*ssa*-complemented strain	This study
HKU16Δ*ssa*(C26S)	HKU16Δ*ssa*::*ssa*-complemented strain containing a C26S substitution	This study
HKU16Δ*speC*	HKU16Δ*speC* isogenic mutant strain	This study
HKU16Δ*spd1*	HKU16Δ*spd1* isogenic mutant strain	This study
HKU16Δ*spd1*++	HKU16Δ*spd1*::*spd1*-complemented strain	This study
HKU16Δ*ssa*/*speC*	HKU16Δ*ssa*/*speC* double isogenic mutant strain	This study
HKU16Δ*ssa*/*spd1*	HKU16Δ*ssa*/*spd1* double isogenic mutant strain	This study
HKU16Δ*speC*/*spd1*	HKU16Δ*speC*/*spd1* double isogenic mutant strain	This study
HKU16Δ*ssa*/*speC*/*spd1*	HKU16Δ*ssa*/*speC*/*spd1* triple isogenic mutant strain	This study
HKU16Δ*ssa*/*speC*/*spd1*++	HKU16Δ*ssa*/*speC*/*spd1*::*ssa*/*speC*/*spd1* complemented strain	This study
HKU16Δ*slo*	HKU16Δ*slo* isogenic mutant strain	This study
HKU16Δ*slo/speC/spd1*	HKU16Δ*slo*/*speC*/*spd1* triple isogenic mutant strain	This study
HKU16Δ*slo/ssa/speC/spd1*	HKU16Δ*slo*/ssa/*speC*/*spd1* quadruple isogenic mutant strain	This study
HKU16-GFP	HKU16 carrying *gfp* reporter pLZ12Km2-P23R:TA:GFP	This study
HKU16Δ*spd1*-GFP	HKU16Δ*spd1* carrying *gfp* reporter pLZ12Km2-P23R:TA:GFP	This study
HKU16Δ*spd1*++-GFP	HKU16Δ*spd1*::*spd1*-complemented strain carrying *gfp* reporter pLZ12Km2-P23R:TA:GFP	This study
**Plasmids**		
Expression plasmids		
pET-28a	Expression plasmid::kanamycin^{R}	Novagen
pET-28a-Spd1	pET-28a+Spd1 expression construct	This study
pET-28a-Spd1_N145A	pET-28a+Spd1 expression construct containing a N145A substitution	This study
pET-151	Directional TOPO expression plasmid, ampicillin^{R}	Invitrogen
pET-151-SSA_N20D/N23A/Y89A/Y94A	pET-151+SSA expression construct containing N20D/N23A/Y89A/Y94A substitutions	This study
pET-15b-SLO	pET-15b+SLO expression construct	[8]
pET-15b-SLOmut	pET-15b+SLO expression construct containing P427L/W535A substitutions	[9]
pET-41a	Expression plasmid:: kanamycin^{R}	Novagen
pET-41a-SSA	pET-41a+SSA expression construct	This study
pET-41a-SSA_C26S	pET-41a+SSA expression construct containing a C26S substitution	This study

pET-41a-SpeC	pET-41a+SpeC expression construct	10
Mutagenesis plasmids		
pLZts	Temperature-sensitive shuttle plasmid, spectinomycin^{R}	11
pLZts-*ssa*_KO	pLZts+*ssa* knockout construct	This study
pLZts-*ssa*_complemented	pLZts+*ssa* complementation construct	This study
pLZts-*ssa*_complemented_C26S	pLZts+*ssa* complementation construct containing a C26S substitution	This study
pLZts-*speC*_KO	pLZts+*speC* knockout construct	This study
pLZts-*spd1*_KO	pLZts+*spd1* knockout construct	This study
pLZts-*spd1*_complemented	pLZts+*spd*1 complementation construct	This study
pLZts-*ssa*/*speC*_KO	pLZts+*ssa*/*speC* double knockout construct	This study
pLZts-*ssa*/*spd1*_KO	pLZts+*ssa*/*spd1* double knockout construct	This study
pLZts-*speC*/*spd1*_KO	pLZts+*speC*/*spd1* double knockout construct	This study
pLZts-*ssa*/*speC*/*spd1*_KO	pLZts+*ssa*/*speC*/*spd1* triple knockout construct	This study
pLZts-*ssa*/*speC*/*spd1*_complemented	pLZts+*ssa*/*speC*/*spd1* complementation construct	This study
pLZts-*slo*_KO	pLZts+*slo* knockout construct	This study
Reporter plasmids		
pLZ12Km2-P23R-TA:GFP	Plasmid-based GFP reporter system	This study

**Primer Name**	**Sequence (5'–3')**
Primers for protein expression constructs	
**Spd1**	
NdeI_*spd1*_SS_pET28a_F	taacatatgatgaaattatctaaacaaaaggcaagtttgcttac
HindIIIstop_*spd1*_pET28a_R	ctgaagcttttattagtttttaggagtggcagttccatttaaatag
*spd1*_N145A_F	cctgaataagcacctgtggctagccaggctgtcattgcc
*spd1*_N145A_R	ggcaatgacagcctggctagccacaggtgcttattcagg
**SSA**	
NcoI_pET-41a_*ssa*_F	gcgccatggcaagtagtcagcctgaccctact
BamHI_pET-41a_*ssa*_R	taagggatccttatttttggtaaggtgaac
*ssa*_C26S_t154a_F	ctacaaaatggttatcatataaacttctcaaattacccataacaccagt
*ssa*_C26S_t154a_R	actggtgttatgggtaatttgagaagtttatatgataaccattttgtag
**SpeC**	
*Nco*I_pET-41a_*speC*_F	cccatggcagactctaagaaagacatttcgaatg
*Bam*HI_pET-41a_*speC*_R	cccggatccttatttttcaagataaatatcgaaatg
Primers for HKU16 gene chromosomal deletion and complementation constructs	
ssa	
*ssa*_KO-S-F	ttggtcgtcagactgatgggcccatgctacaaggggagagaatc
*ssa*_KO-S-R	gaacctctatgagtattcttattcttttattcatttggctacctcttatatatttaaaac
*ssa*_KO-AS-F	aagaatactcatagaggttcaccttaccaaaaaataaaagaaaataac
ssa_KO-AS-R	cataacctgaaggaagatctcttaaataacaaaattattctagaaaaagatatcg

speC	
*speC*_KO-S-F	ttggtcgtcagactgatgggccccactaaaataaattatgaccctg
*speC*_KO-S-R	tatcgaaatgtttgatgatgttaatctttttcattttttc
*speC*_KO-AS-F	catcatcaaacatttcgatatttatcttgaaaaataattc
*speC*_KO-AS-R	cataacctgaaggaagatctgtttgatattacaactaataaaaaacaag
spdI	
*spd1*_KO-S-F	ttggtcgtcagactgatgggcccaacaaaaaagaaccttaatatgg
*spd1*_KO-S-R	cagttccatttgccttttgtttagataatttc
*spd1*_KO-AS-F	acaaaaggcaaatggaactgccactcctaaaaac
*spd1*_KO-AS-R	cataacctgaaggaagatctttttaaagtcaatttcctggaaagttac
slo	
*slo*_KO-S-F	ttggtcgtcagactgatgggcccggtgaccataaaaaagtaac
*slo*_KO-S-R	tcgaaccatattttttgttagacatgtccttc
*slo*_KO-AS-F	ctaacaaaaaatatggttcgattacttataagtag
*slo*_KO-AS-R	cataacctgaaggaagatctgagatttccagccttcattataac
ssa*/*speC[1]	
*ssa_speC*_KO-S-F	ttggtcgtcagactgatgggcccattcataggataatcacactag
*ssa_speC*_KO-S-R	taacatcatcaaacatttcgatatttatcttgaaaaataattc
*speC*_KO-S-R	tatcgaaatgtttgatgatgttaatctttttcattttttc
*ssa_speC*_KO-AS-R	cataacctgaaggaagatctctaccttttcacatatccaac
ssa*/*spd1[1]	
*ssa_spd1*_KO-S-F	ttggtcgtcagactgatgggccctaaagtcaatttcctggaaagttac
*ssa_spd1*_KO-S-R	acaaaaggcaggaactgccactcctaaaaac
*ssa_spd1*_KO-AS-F	tggcagttcctgccttttgtttagataatttc
*ssa_spd1*_KO-AS-R	cataacctgaaggaagatctatcatttgctatcattgcc
speC*/*spd1	
*speC_spd1*_KO-S-F	ttggtcgtcagactgatgggccctcaataattctccgtacgag
*speC_spd1*_KO-S-R	acaaaaggcacatttcgatatttatcttgaaaaataattc
*speC_spd1*_KO-AS-F	tatcgaaatgtgccttttgtttagataatttc
*ssa_spd1*_KO-AS-R	cataacctgaaggaagatctatcatttgctatcattgcc
ssa*/*speC*/*spd1[1]	
*ssa_speC*_KO-S-F	ttggtcgtcagactgatgggcccattcataggataatcacactag
*speC_spd1*_KO-S-R	acaaaaggcacatttcgatatttatcttgaaaaataattc
*speC_spd1*_KO-AS-F	tatcgaaatgtgccttttgtttagataatttc
*ssa_spd1*_KO-AS-R	cataacctgaaggaagatctatcatttgctatcattgcc
Primers for quantitative real time PCR	
qRTPCR-*gyrA*-F	cgacttgtctgaacgccaaa
qRTPCR-*gyrA*-R	gtcagcaatcaaggccaaca
qRTPCR-*ssa*-F	gcctgaccctactccagaac

qRTPCR-*ssa*-R	agctgacctgtggatcttaca
qRTPCR-*speC*-F	ccgaaatgtcttatgaggcctc
qRTPCR-*speC*-R	agcaggcgtaattcctccat
Mouse genotyping primers	
HLA-DR4-DQ8-F	tcccttgatgatgaagatgg
HLA-DR4-DQ8-R	cagaggtaactgtgctcacg
hCD4-F	ctttccagaaggcctccagca
hCD4-R	ctctcatcaccaccaggttcac

[1]HKU16Δ*ssa* genomic DNA served as template and HKU16Δ*ssa* was used for genetic manipulation.

References

1 Loh, J. M. & Proft, T. Toxin-antitoxin-stabilized reporter plasmids for biophotonic imaging of group A *Streptococcus*. *Appl Microbiol Biotechnol* **97**, 9737-9745 (2013).

2 Ly, D. *et al.* Plasmin(ogen) acquisition by group A *Streptococcus* protects against C3b-mediated neutrophil killing. *J Innate Immun* **6**, 240-250 (2014).

3 Sundberg, E. & Jardetzky, T. S. Structural basis for HLA-DQ binding by the streptococcal superantigen SSA. *Nat Struct Biol* **6**, 123-129 (1999).

4 Lee, K. H., Wucherpfennig, K. W. & Wiley, D. C. Structure of a human insulin peptide-HLA-DQ8 complex and susceptibility to type 1 diabetes. *Nat Immunol* **2**, 501-507 (2001).

5 Jardetzky, T. S. *et al.* Three-dimensional structure of a human class II histocompatibility molecule complexed with superantigen. *Nature* **368**, 711-718 (1994).

6 Wertman, K. F., Wyman, A. R. & Botstein, D. Host/vector interactions which affect the viability of recombinant phage lambda clones. *Gene* **49**, 253-262 (1986).

7 Tse, H. *et al.* Molecular characterization of the 2011 Hong Kong scarlet fever outbreak. *J Infect Dis* **206**, 341-351 (2012)

8 Timmer, A. M. *et al.* Streptolysin O promotes group A *Streptococcus* immune evasion by accelerated macrophage apoptosis. *J Biol Chem* **284**, 862-871 (2009).

9 Rivera-Hernandez, T. *et al.* Differing efficacies of lead group A streptococcal vaccine candidates and full-length M protein in cutaneous and invasive disease models. *mBio* **7**, pii: e00618-16 (2016).

10 Kasper, K. J. *et al.* Bacterial superantigens promote acute nasopharyngeal infection by *Streptococcus pyogenes* in a human MHC Class II-dependent manner. *PLoS Pathog* **10**, e1004155-e1004155 (2014).

11 Barnett, T. C., Daw, J. N., Walker, M. J. & Brouwer, S. Genetic manipulation of group A *Streptococcus* – gene deletion by allelic replacement. *In: T. Proft and J. Loh (Ed) Group A Streptococcus: Methods and Protocols. Springer, Heidelberg.*, 59-69 (2020).
