# Supplementary material for: Prophage exotoxins enhance colonization fitness in epidemic scarlet fever-causing Streptococcus pyogenes
Source: Nat Commun. 2020 Oct 6;11:5018. doi: 10.1038/s41467-020-18700-5 (PMC7538557; doi:10.1038/s41467-020-18700-5)
Supplement: Supplementary file 2 — Reporting Summary [file 41467_2020_18700_MOESM2_ESM.pdf]

nature research

Corresponding author(s): Mark Walker

Last updated by author(s): Sep 1, 2020

Reporting Summary

Nature Research wishes to improve the reproducibility of the work that we publish. This form provides structure for consistency and transparency in reporting. For further information on Nature Research policies, see our Editorial Policies and the Editorial Policy Checklist.

Statistics

For all statistical analyses, confirm that the following items are present in the figure legend, table legend, main text, or Methods section.

n/a	Confirmed	
☐	☒	The exact sample size (*n*) for each experimental group/condition, given as a discrete number and unit of measurement
☐	☒	A statement on whether measurements were taken from distinct samples or whether the same sample was measured repeatedly
☐	☒	The statistical test(s) used AND whether they are one- or two-sided<br>*Only common tests should be described solely by name; describe more complex techniques in the Methods section.*
☒	☐	A description of all covariates tested
☒	☐	A description of any assumptions or corrections, such as tests of normality and adjustment for multiple comparisons
☐	☒	A full description of the statistical parameters including central tendency (e.g. means) or other basic estimates (e.g. regression coefficient) AND variation (e.g. standard deviation) or associated estimates of uncertainty (e.g. confidence intervals)
☐	☒	For null hypothesis testing, the test statistic (e.g. *F*, *t*, *r*) with confidence intervals, effect sizes, degrees of freedom and *P* value noted<br>*Give P values as exact values whenever suitable.*
☒	☐	For Bayesian analysis, information on the choice of priors and Markov chain Monte Carlo settings
☒	☐	For hierarchical and complex designs, identification of the appropriate level for tests and full reporting of outcomes
☒	☐	Estimates of effect sizes (e.g. Cohen's *d*, Pearson's *r*), indicating how they were calculated

Our web collection on statistics for biologists contains articles on many of the points above.

Software and code

Policy information about availability of computer code

Data collection	No software was used for data collection.

Data analysis

Data analysis was performed using the following software:

BWA MEM (version 0.7.16), available at https://github.com/lh3/bwa/releases

DESeq2 (version 1.26.0), available at https://bioconductor.org/packages/release/bioc/html/DESeq2.html

R (version 3.6.0), available at https://www.r-project.org/

Integrative Genomics Viewer (IGV2.4.5), available at http://software.broadinstitute.org/software/igv/

ggplot2 (version 3.2.1), available at https://github.com/tidyverse/ggplot2

ImageJ software (version 1.8.0), available at https://imagej.nih.gov/ij/download.html

CellProfiler (version 3.1.9), available at https://github.com/CellProfiler/CellProfiler

Prism8 (version 8.4.3), available at https://www.graphpad.com/scientific-software/prism/

Quant Studio Real-Time PCR software v1.1 (QuantStudio 6 Flex, Life Technologies), available at https://www.thermofisher.com/au/en/home/global/forms/life-science/quantstudio-6-7-flex-software.html

For manuscripts utilizing custom algorithms or software that are central to the research but not yet described in published literature, software must be made available to editors and reviewers. We strongly encourage code deposition in a community repository (e.g. GitHub). See the Nature Research guidelines for submitting code & software for further information.

Data

Policy information about availability of data

All manuscripts must include a data availability statement. This statement should provide the following information, where applicable:

- Accession codes, unique identifiers, or web links for publicly available datasets
- A list of figures that have associated raw data
- A description of any restrictions on data availability

Data supporting the findings of this study are available within the paper and its supplementary information files, or are available from the authors upon request.

Illumina read data are available on NCBI under the sample accession numbers relating to the three conditions (in triplicate): THY (ERS1091539, ERS1091548, ERS1091557); THY plus erythromycin (ERS1091542, ERS1091551, ERS1091560); THY plus mitomycin C (ERS1091545, ERS1091554, ERS1091563).

Field-specific reporting

Please select the one below that is the best fit for your research. If you are not sure, read the appropriate sections before making your selection.

☒ Life sciences ☐ Behavioural & social sciences ☐ Ecological, evolutionary & environmental sciences

For a reference copy of the document with all sections, see nature.com/documents/nr-reporting-summary-flat.pdf

Life sciences study design

All studies must disclose on these points even when the disclosure is negative.

Sample size

Sample sizes were not predetermined based on statistical methods, but were chosen according to the standards in the field - at least 3 independent biological replicates for each condition. This generated sufficient data for statistical analysis. For animal studies, sample sizes were chosen according to the following publications:

Kasper, K. J. et al. Bacterial superantigens promote acute nasopharyngeal infection by Streptococcus pyogenes in a human MHC Class II-dependent manner. PLoS Pathog 10, e1004155 (2014).

Zeppa, J. J. et al. Nasopharyngeal infection by Streptococcus pyogenes requires superantigen-responsive Vbeta-specific T cells. Proc Natl Acad Sci USA 114, 10226–10231 (2017).

Data exclusions

No data were excluded from the analyses.

Replication

Numbers of experimental replicates are stated in each figure legend. Reported results were consistently replicated across multiple experiments with all replicates generating similar results.

Randomization

No randomization was necessary as experiments were performed with appropriate controls. Randomization is not generally used in this field. For animal studies, mice were sex- and aged-matched to take into account these possible variables in response magnitudes.

Blinding	Investigators were not blinded. Blinding during analysis was not necessary because the results are quantitative and did not require subjective judgment or interpretation. Blinding is not typically used in the field.

Reporting for specific materials, systems and methods

We require information from authors about some types of materials, experimental systems and methods used in many studies. Here, indicate whether each material, system or method listed is relevant to your study. If you are not sure if a list item applies to your research, read the appropriate section before selecting a response.

Materials & experimental systems

n/a	Involved in the study	
☐	☒	Antibodies
☐	☒	Eukaryotic cell lines
☒	☐	Palaeontology and archaeology
☐	☒	Animals and other organisms
☐	☒	Human research participants
☒	☐	Clinical data
☒	☐	Dual use research of concern

Methods

n/a	Involved in the study	
☒	☐	ChIP-seq
☒	☐	Flow cytometry
☒	☐	MRI-based neuroimaging

Antibodies

Antibodies used	Affinity-purified rabbit antibody to SpeC was purchased from Toxin Technology (catalogue number PCI333; 1:1,000 dilution). Affinity-purified rabbit antibody to SSA was produced by Mimotopes, Clayton, Australia raised against the peptide H-CGGSSQPDPTPEQLNKSSQFTG-OH coupled to Keyhole Limpet Hemocyanin (1:500 dilution). Mouse polyclonal antibodies to Spd1 (1:1,000 dilution) and SLO (1:2,000 dilution) were generated in this study as described in the methods section. Affinity-purified rabbit antibody to SpeB was purchased from Toxin Technology (catalogue number PBI222; 1:1,000 dilution). Anti-rabbit IgG (H+L) (DyLight™ 800 4X PEG Conjugate, NEB, 5151P; 1:10,000 dilution) and anti-mouse IgG (H+L) (DyLight™ 800 4X PEG Conjugate, NEB, 5257S; 1:10,000 dilution) were used as secondary antibodies.
Validation	Specificity of each primary antibody was validated by Western Blotting using purified recombinant protein and culture supernatants of respective isogenic mutant strains of Streptococcus pyogenes. Detection with secondary antibodies validated the species-specific source of each primary antibody.

Eukaryotic cell lines

Policy information about cell lines

Cell line source(s)	The human nasopharyngeal carcinoma epithelial cell line Detroit 562 used in this study was purchased from the ATCC cell biology collection (ATCC CCL-138, Lot 70004014).
Authentication	Certificate of Analysis was obtained from ATCC. The cell line was not authenticated.
Mycoplasma contamination	The cell line Detroit 562 tested negative for mycoplasma contamination in the Certificate of Analysis. No further testing for mycoplasma contamination has been carried out.
Commonly misidentified lines (See ICLAC register)	No commonly misidentified cell lines were used in the study.

Animals and other organisms

Policy information about studies involving animals; ARRIVE guidelines recommended for reporting animal research

Laboratory animals	Mice were bred and housed at the University of Queensland's Australian Institute for Bioengineering and Nanotechnology (AIBN) (Brisbane, Queensland, Australia). Transgenic C57BL/6J mice expressing human major histocompatibility complex II molecules DR4/DQ8 and human CD4 (HLA-B6) were kindly provided by James McCluskey (University of Melbourne, Melbourne, Australia). The genotype was determined by tail DNA PCR using the following primers: HLA-DR4-DQ8-F (5'-tcccttgatgatgaagatgg-3'), HLA-DR4-DQ8-R (5'-cagaggtaactgtgctcacg-3'), hCD4-F (5'-ctttccagaaggcctccagc-3') and hCD4-R (5'-ctctcatcaccaccaggttcac-3'). Gender- and age-matched (9- to 13-week-old) HLA-B6 mice were used in the study. For immunization studies, 4 - 6 weeks old BALB/c mice were purchased from the Animal Resources Centre (PO Box 1180, Canning Vale, Western Australia, 6970 Australia).
Wild animals	No wild animals were used in the study.
Field-collected samples	No field-collected samples were used in the study.
Ethics oversight	Animal experiments were performed according to the Australian code of practice for the care and use of animals for scientific purposes. Permission was obtained from the University of Queensland ethics committee to undertake this work (SCMB/140/16/NHMRC).

Note that full information on the approval of the study protocol must also be provided in the manuscript.

Human research participants

Policy information about studies involving human research participants

Population characteristics	Human blood was sourced from healthy volunteers in Ontario (Canada), Brisbane (Australia) and Wollongong (Australia) who were willing and deemed eligible to donate blood. Blood from both females and males was drawn from participants aged 20 to 65 years old.
Recruitment	All participation was voluntary and participants were volunteers from the laboratory itself and usually directly involved in the experiment. Recruitment was done by word of mouth (eg. volunteers from surrounding laboratories) or institute emailing lists and a consent form was signed. Different donors were selected from three geographical locations.
Ethics oversight	The Health Sciences Research Ethics Board at Western University (Ontario, Canada) (Protocol #110859)<br><br>The University of Queensland medical research ethics committee (2010001586)<br><br>The University of Wollongong Human Research Ethics Committee (HE08/250)

Note that full information on the approval of the study protocol must also be provided in the manuscript.
